# Chimeric Antigen Receptor T Cells Targeting Integrin αvβ6 Expressed on Cholangiocarcinoma Cells

**DOI:** 10.3389/fonc.2021.657868

**Published:** 2021-03-03

**Authors:** Nattaporn Phanthaphol, Chalermchai Somboonpatarakun, Kwanpirom Suwanchiwasiri, Thaweesak Chieochansin, Jatuporn Sujjitjoon, Sopit Wongkham, John Maher, Mutita Junking, Pa-thai Yenchitsomanus

**Affiliations:** ^1^Graduate Program in Immunology, Department of Immunology, Faculty of Medicine Siriraj Hospital, Mahidol University, Bangkok, Thailand; ^2^Siriraj Center of Research Excellence for Cancer Immunotherapy (SiCORE-CIT), Faculty of Medicine Siriraj Hospital, Mahidol University, Bangkok, Thailand; ^3^Division of Molecular Medicine, Research Department, Faculty of Medicine Siriraj Hospital, Mahidol University, Bangkok, Thailand; ^4^Molecular Medicine Program, Multidisciplinary Unit, Faculty of Science, Mahidol University, Bangkok, Thailand; ^5^Department of Biochemistry, Faculty of Medicine, Khon Kaen University, Khon Kaen, Thailand; ^6^Cholangiocarcinoma Research Institute, Khon Kaen University, Khon Kaen, Thailand; ^7^King's College London, King's Health Partners Integrated Cancer Centre and Division of Cancer Studies, Guy's Hospital, London, United Kingdom

**Keywords:** adoptive T cell therapy, chimeric antigen receptor, integrin αvβ6, immunotherapy, cholangiocarcinoma

## Abstract

Cholangiocarcinoma (CCA) is a lethal bile duct cancer that responds poorly to current standard treatments. A new therapeutic approach is, therefore, urgently needed. Adoptive T cell transfer using chimeric antigen receptor (CAR) T cells is a new therapeutic modality with demonstrated efficacy in hematologic malignancies. However, its efficacy against solid tumors is modest, and further intensive investigation continues. An important factor that influences the success of CAR T cell therapy is the selection of a target antigen that is highly expressed on cancer cells, but markedly less so in normal cells. Integrin αvβ6 is upregulated in several solid tumors, but is minimally expressed in normal epithelial cells, which suggests integrin αvβ6 as an attractive target antigen for CAR T cell immunotherapy in CCA. We investigated integrin αvβ6 expression in pathological tissue samples from patients with liver fluke-associated CCA. We then created CAR T cells targeting integrin αvβ6 and evaluated their anti-tumor activities against CCA cells. We found overexpression of the integrin αvβ6 protein in 23 of 30 (73.3%) CCA patient tissue samples. Significant association between high integrin αvβ6 expression and short survival time (*p* = 0.043) was also observed. Lentiviral constructs were engineered to encode CARs containing an integrin αvβ6-binding peptide (A20) derived from foot-and-mouth disease virus fused with a second-generation CD28/CD3ζ signaling domain (A20-2G CAR) or with a fourth-generation CD28/4-1BB/CD27/CD3ζ signaling domain (A20-4G CAR). The A20-2G and A20-4G CARs were highly expressed in primary human T cells transduced with the engineered lentiviruses, and they exhibited high levels of cytotoxicity against integrin αvβ6-positive CCA cells (*p* < 0.05). Interestingly, the A20-2G and A20-4G CAR T cells displayed anti-tumor function against integrin αvβ6-positive CCA tumor spheroids (*p* < 0.05). Upon specific antigen recognition, A20-4G CAR T cells produced a slightly lower level of IFN-γ, but exhibited higher proliferation than A20-2G CAR T cells. Thus, the A20-4G CAR T cells with lower level of cytokine production, but with higher proliferation represents a promising potential adoptive T cell therapy for integrin αvβ6-positive CCA.

## Introduction

Cholangiocarcinoma (CCA) arises from the cancerous transformation of epithelial cells lining the bile duct. It is a relatively rare cancer, but its rates of both incidence and mortality are increasing worldwide (1). The highest incidence of CCA was found in the Northeast of Thailand where infection with an oncogenic liver fluke [*Opisthorchis viverrini* (OV)] is known to be a strong risk factor for CCA (2). Surgical resection is a curative treatment for CCA; however, only 20-40% of tumors are resectable, and the recurrence rate after surgery is high (3). For non-resectable patients, the standard first-line therapy is gemcitabine in combination with cisplatin. However, this therapeutic regimen achieves a 5-year overall survival rate of <5%, and the median overall survival is <1 year (4). Immunotherapy and targeted therapy for this difficult-to-treat disease have been reported (5, 6); however, the limited efficacy of these therapies highlights the need for an alternative treatment approach.

Generally, CCA and other cancers develop when transformed cells escape immune surveillance. Downregulation of MHC molecules that conceal cancerous cells from T cell recognition is one of many cancer immune escape mechanisms (7, 8). To overcome this problem in cancer treatment, adoptive transfer of T cells expressing a chimeric antigen receptor (CAR) has been developed as a promising therapeutic approach. CARs are synthetic receptors that mimic natural T cell receptor function by combining a cancer antigen-binding domain with a T cell activating signaling domain. CAR T cells recognize cancer antigen in a direct, antibody-like fashion, which leads to the activation of intracellular signaling. As a result, CAR T cells kill cancerous cells in an MHC-independent manner.

Different generations of CAR T cells have been developed by combining the intracellular part of T cell receptor (CD3ζ) and one or more co-stimulatory domains. Recently, three second-generation CAR (2G-CAR) T cells targeting CD19 for hematologic malignancies were approved by the U.S. Food and Drug Administration (USFDA), namely Kymriah (4-1BB/CD3), Yescarta, and Tecartus (CD28/CD3ζ). However, clinically successful CAR T cell therapies in patients with solid tumors have been limited, and studies to improve the efficacies of these therapies are intensively ongoing. Several research groups have designed other generations of CAR T cells by adding more co-stimulatory domains into the CAR molecule (9, 10). Third-generation CAR (3G CAR) T cells consisting of CD28/CD137/CD3ζ (11, 12) or CD28/CD27/CD3ζ (13) were created and tested. Fourth-generation CAR (4G CAR) T cells containing CD28/CD137/CD27/CD3ζ have also been produced and proven effective in the treatment of B cell leukemias (10, 14, 15).

An essential factor that influences the success of CAR T cell immunotherapy is the selection of a target antigen that is highly expressed on the surface of cancerous cells, but that is only minimally expressed on normal cells. Binding between the target antigen on cancerous cells and the extracellular antigen-binding domain of the CAR molecule leads to activation of CAR T cells to kill cancerous cells. An attractive potential target antigen in solid tumors is integrin αvβ6 because it is overexpressed in multiple epithelial malignancies, including pancreatic ductal adenocarcinoma (16), ovarian cancer (17), head and neck squamous cell carcinoma (18, 19), breast cancer (20), and CCA (21, 22). Integrin αvβ6 is also a target for diagnostic imaging and anti-cancer therapies (19). Several integrin αvβ6-specific therapeutic agents have been studied in clinical trials (23–25). Notably, the specificity of integrin αvβ6 immunohistochemistry for CCA (100%) surpassed all other tested markers, and the sensitivity was very similar to that of cytokeratin 7 (CK7) (86 vs. 90%) (26). Integrin αvβ6 is, thus, a potential target antigen for development of CAR T cell therapy for CCA.

A single-chain variable fragment (scFv) derived from a monoclonal antibody is most commonly used as the extracellular cancer antigen-binding domain within tumor-specific CAR molecules. However, antigens on cancer cells can also be specifically bound by the peptide with high specificity for the desired target (27). Second-generation (2G) of CAR T cells targeting integrin αvβ6 were recently tested in multiple solid tumors, including pancreatic, breast, and ovarian cancers (28). The antigen-binding domain of this 2G CAR was a peptide containing 20-mers of amino acids (A20) that was derived from the viral capsid protein 1 (VP1) of foot-and-mouth disease virus (FMDV), and it bound explicitly with high affinity (<1 nM) only to integrin αvβ6 (29). However, the effect of CAR T cells targeting integrin αvβ6 in the killing of CCA cells is still unknown.

Expression of integrin αvβ6 has not been previously characterized in CCA tumors or cell lines from patients with liver fluke-associated CCA. To evaluate its potential as a therapeutic target antigen in CCA, we analyzed integrin αvβ6 expression in pathological tissue samples and CCA tumor cell lines. We then compared the anti-tumor activity of A20-2G CAR T cells and A20-4G CAR T cells, containing the CD28/CD3ζ and CD28/4-1BB/CD27/CD3ζ signaling domains, respectively. Both CARs demonstrated integrin αvβ6-dependent anti-tumor activity in models of CCA, but the same anti-tumor activity was not observed in non-transduced (NT) T cells. The results of this study provide proof-of-principle for the use of integrin αvβ6-specific CAR T cells in adoptive T cell immunotherapy of CCA.

## Materials and Methods

### Ethical Approval

In this study, paraffin-embedded tissues from patients with CCA were collected at Srinagarind Hospital, Khon Kaen University, Khon Kaen, Thailand. The protocols for collection of the tissue samples and the experimental studies were approved by the Ethics Committee for Human Research, Khon Kaen University (No. HE591063). Written informed consent was obtained from each patient before the study. Peripheral blood mononuclear cells (PBMCs) were obtained from healthy volunteers who had provided written informed consent in accordance with a protocol approved by the Siriraj Institutional Review Board of the Faculty of Medicine Siriraj Hospital, Mahidol University, Bangkok, Thailand (COA No. *Si* 829/2020).

### Immunohistochemistry

Integrin αvβ6 was detected on the formalin-fixed, paraffin-embedded tissue sections using immunohistochemistry (IHC). Specifically, 4-μm-thick paraffin sections were deparaffinized in xylene, and then rehydrated in gradient ethanol. These tissue sections were antigen-retrieved in 0.1% trypsin, and endogenous peroxidases were blocked in 3% H_2_O_2_. After blocking of non-specific binding with 5% skim milk, the tissue sections were incubated overnight with an anti-αvβ6 monoclonal antibody (dilution 1:100, Clone 6.2A1; Biogen Inc., Cambridge, MA, USA) that was generously provided by Dr. Shelia M. Violette. The treated tissue sections were then reacted with EnVision Kit/Horseradish Peroxidase (HRP)^TM^ (Agilent Technologies, Santa Clara, CA, USA) at room temperature (RT) for 1 h. Immunoreactive integrin αvβ6 from those tissue sections were developed using 3, 3′-diaminobenzidine (DAB) solution. The tissue sections were subsequently counterstained with Mayer's Hematoxylin (Sigma-Aldrich Corporation, St. Louis, MO. USA), mounted, and observed under a light microscope (Nikon Eclipse Ti2; Nikon Instruments, Tokyo, Japan).

The IHC-stained tissues were evaluated independently by two investigators who had no prior knowledge of patient clinical or survival data. The frequency of integrin αvβ6 was semi-quantitatively scored according to the percentage of positive cells, as follows: 0%, negative; 1-25%, +1; 26-50%, +2; and, 50%, +3. The intensity of protein staining was scored, as follows: weak, 1; moderate, 2; and, strong, 3. The expression of integrin αvβ6 was evaluated using H-score by multiplying the frequencies and intensities. The patients were then categorized into two groups according to the median IHC score. Log-rank test was used to analyze the difference between these two groups relative to overall survival.

### Cell Culture

Human CCA cell lines, KKU-055 (JCRB1551), KKU-100 (JCRB1568) (29), and KKU-213A (JCRB1557) (30), were established from patients with CCA in opisthorchiasis endemic areas located in the Northeastern region of Thailand. The Lenti-X 293T cell line is a highly transfectable subclone of the transformed human embryonic kidney cell line HEK 293, which supports high levels of viral production (Takara Bio, Kusatsu, Shiga, Japan). A375.puro (αvβ6-negative) and A375.β6 (αvβ6-positive) cell lines were used a negative and positive controls, respectively (28, 31). Tumor cell lines were cultured in Dulbecco's Modified Eagle's Medium (DMEM) or in DMEM/F12 (Gibco; Invitrogen Corporation, Carlsbad, CA, USA) supplemented with 10% fetal bovine serum (FBS), penicillin (100 U/ml), and streptomycin (0.1 mg/ml) at 37°C with 5% CO_2_.

### Immunofluorescence Staining

Cells were cultured on glass coverslips and fixed with 4% paraformaldehyde for 15 min on ice. The cells were then washed with phosphate-buffered saline (PBS) before incubation with 5% bovine serum albumin (BSA) blocking solution for 30 min. The cells were then incubated with a 1:100 dilution of anti-β6 polyclonal antibody (clone ITBG6; Thermo Fisher Scientific, Waltham, MA, USA) at 4°C overnight. After washing, the cells were incubated with anti-mouse Alexa Fluor® 488-labeled (clone A21206; Thermo Fisher Scientific), and their nuclei were counterstained with Hoechst 33342 dye for 1 h at RT. The coverslips were mounted on a glass slide and immunofluorescence signals were visualized using a Zeiss LSM 800 confocal microscope (Carl Zeiss Microscopy, Jena, Germany).

### Flow Cytometry

Integrin αvβ6 expression on the surface of CCA cells was detected using mouse anti-integrin αvβ6 antibody (Clone 10D5; Merck Millipore, Burlington, MA, USA) at a dilution of 1:100. The cells were then washed and incubated with Alexa Fluor® 488-tagged secondary antibody (Thermo Fisher Scientific). CAR expression on transduced T cells was detected using anti-cMyc FIT-C-tagged antibody (clone ab1394; Abcam, Cambridge, UK). Phenotypic analysis of T cells was performed by using anti-CD3-FITC (Clone UCHT-1), anti-CD4-APC (Clone MEM-241), anti-CD8-APC (Clone UCHT-4), anti-CD16-APC (Clone 3G8), and anti-CD56-PE (Clone AB_2563925). All of these antibodies were purchased from BioLegend (San Diego, CA, USA). Flow cytometry was performed using a BD Accuri C6 Plus Flow Cytometer (BD Biosciences, San Jose, CA, USA), and the data was analyzed by using FlowJo software (FlowJo LLC, Ashland, OR, USA).

### Construction of Chimeric Antigen Receptor

Second-generation CAR T cells containing A20 peptide for targeting integrin αvβ6 (A20-2G) CAR T cells were created as previously described (28). Briefly, A20 peptide derived from FMDV was placed downstream of a CD124 signal sequence, followed by human *c-myc* peptide tag (EQKLISEEDL), as shown in **Figure 3A**. The DNA fragment encoding the required parts was synthesized by Integrated DNA Technologies (Coralville, IA, USA). The A20 codon-optimized gene was sub-cloned into self-inactivating lentivirus vectors (pCDH) containing expression cassettes encoding CD8 short hinge, a CD28 transmembrane domain, and the CD28/CD3ζ (A20-2G) or CD28/4-1BB/CD27/CD3ζ (A20-4G) signaling domains. Transgene expression is driven by the elongation factor-1α (EF-1α) promoter. Plasmid DNA was isolated using a Midiprep Kit (Qiagen, Hilden, Germany) and sequences were verified by DNA sequencing.

### Lentivirus Production and T Cell Transduction

Lenti-X 293T cells were transfected with the A20-2G or A20-4G plasmid and two packaging plasmids (psPAX2 and pMD.2G) at a ratio of 5:3:1 using calcium phosphate transfection method. The supernatant was collected at 48 and 72 h post-transfection and filtered through a 0.45 μm filter unit (Merck Millipore) to remove cell debris, followed by concentration at 20,000 × g for 2 h at 4°C (Sorvall RC-6 Plus Centrifuge; Thermo Fisher Scientific). Virus titer was determined using a qPCR Lentiviral Titration Kit (ABM, Richmond, BC, Canada) according to the manufacturer's instructions.

Peripheral blood mononuclear cells (PBMCs) were isolated from blood samples of healthy donors using density gradient centrifugation, and activated by culturing with 5 μg/ml phytohemagglutinin-L (PHA-L) (Roche Applied Science, Penzberg, Germany) in AIM-V medium supplemented with 5% human serum, IL-2 (20 ng/ml), IL-7 (10 ng/ml), and IL-15 (40 ng/ml) (Immunotools, Friesoythe, Germany). On day 3 after activation, T cells were transduced with lentiviral particles using 10 μg/ml protamine sulfate (Sigma-Aldrich). The cells were centrifuged at 1200 × g at 32°C for 90 min, followed by incubation at 37°C and 5% CO_2_ overnight.

### Immunoblot Analysis

Immunoblotting was used to detect the expression of A20-2G or A20-4G CAR construct following transfection in Lenti-X 293T cells. In brief, the cells were lysed in radioimmunoprecipitation assay (RIPA) lysis buffer. Cell lysate was then resolved by sodium dodecyl sulfate–polyacrylamide gel electrophoresis (SDS-PAGE) and subsequently transferred onto a nitrocellulose membrane. The membrane was blocked with 5% skim-milk in Tris-buffered saline (TBS) and 0.1% Tween-20 (TBS-T), and then detected with anti-CD3ζ (clone sc-166435) and anti-GAPDH (clone sc-32233) from Santa Cruz Biotechnology (Dallas, TX, USA). The membrane was incubated with HRP-conjugated secondary antibody (Invitrogen) and the immunoreaction was developed using chemiluminescence reagents (SuperSignal® West Pico Substrate; Thermo Fisher Scientific). The signal from the reaction was captured on X-ray film and quantified using ImageJ program (National Institutes of Health, Bethesda, MD, USA). The expression level of GAPDH was used as loading control.

### Cytotoxicity Assays

The monolayer of target cells (1 × 10^4^) was co-cultured with A20-2G CAR T cells or A20-4G CAR T cells or NT T cells at three different effector to target (E:T) ratios (5:1, 2.5:1, and 1.25:1) for 24-48 h. After removal of the CAR T cells, 100 μl of crystal violet fixing/staining solution was added to each well and incubated for 20 min. The plates were washed and the cell-bound dye was dissolved in methanol. The absorbance was measured at a wavelength of 595 nm using a Sunrise™ Absorbance Microplate Reader (Magellan™ data analysis software version 6.6.0.1; Tecan, Männedorf, Switzerland). Cytotoxicity was calculated using the following formula: [1-(absorbance of monolayer culture with T cells/absorbance of monolayer culture alone)] × 100%.

In addition, cytotoxicity assay was conducted by using three-dimensional (3D) spheroid model. In brief, a total number of 2 × 10^3^ cancer cells were firstly stained with CellTracker™ Green CMFDA (5-chloromethylfluorescein diacetate) Dye (Thermo Fisher Scientific, Waltham, MA) and then seeded into an ultra-low attachment 96-well round-bottomed plate (Corning, NY, USA) containing 2.5% Corning matrigel matrix (Corning, NY, USA). To generate a single spheroid, the plate was centrifuged at 1,000 × g at 4°C for 10 min and then cultured for 48 h. T cells in culture medium containing 1 μg/ml propidium iodide (PI) were added to the spheroid at E:T ratio of 5:1. After co-culturing for 3 days, dead cancer cells, which were stained with PI, were analyzed by a confocal microscope (Nikon Instruments Inc., Melville, NY, USA). Quantification of mean fluorescence intensity (MFI) was conducted by using NIS-Elements software. Cytotoxicity was calculated by following formula: [(experimental MFI-spontaneous MFI)/(maximum MFI-spontaneous MFI)] × 100. Experimental and spontaneous MFIs were MFI of sample spheroids when co-cultured with or without CAR T cells, respectively. Maximum MFI was MFI of spheroid treated with 0.1% Triton-X 100.

### Intracellular Cytokine Staining and T Cell Proliferation Assay

CAR T cells were cultured with target cells in media containing Brefeldin A (BioLegend) at an E:T ratio of 5:1 for 6 h in 5% CO_2_ at 37°C. The CAR T cells were harvested and the cell surface markers were stained with anti-CD3-FITC and anti-CD8-APC antibodies (Immunotools). The cells were then fixed with 4% paraformaldehyde for 15 min. Intracellular cytokine was stained by incubation with anti-IFN-γ-PE (Immunotools) antibody in the presence of 0.5% saponin permeabilization agent on ice for 30 min. The cells were then subjected to flow cytometry to analyze cytokine production levels.

T cell proliferation was assessed by tracking cells labeled with carboxyfluorescein succinimidyl ester (CFSE). Briefly, 1 × 10^5^ of T cells were stained with 1 μM CFSE for 10 min at 37°C. After washing twice with culture media, CFSE-labeled T cells were co-cultured with target cell monolayers at an E:T ratio of 5:1 in AIM-V medium supplemented with 5% human serum. On day 3 after co-culturing, CFSE dilution was measured by flow cytometry to estimate proliferation of total CAR T cells. Note that no exogenous cytokines were added during the proliferation assay.

### Statistical Analysis

GraphPad Prism 7 software (GraphPad Software, San Diego, CA, USA) was used for statistical analysis. Data are presented as mean ± standard deviation (SD) or standard error of the mean (SEM). For comparison between two groups, a two-tailed *t*-test was used. For comparisons among three or more groups, one-way analysis of variance (ANOVA) with Bonferroni's *post hoc* test was used. A *p*-value < 0.05 was considered statistically significant.

## Results

### Expression of Integrin αvβ6 in Human CCA Tissues and Cell Lines

**Figure 1:**
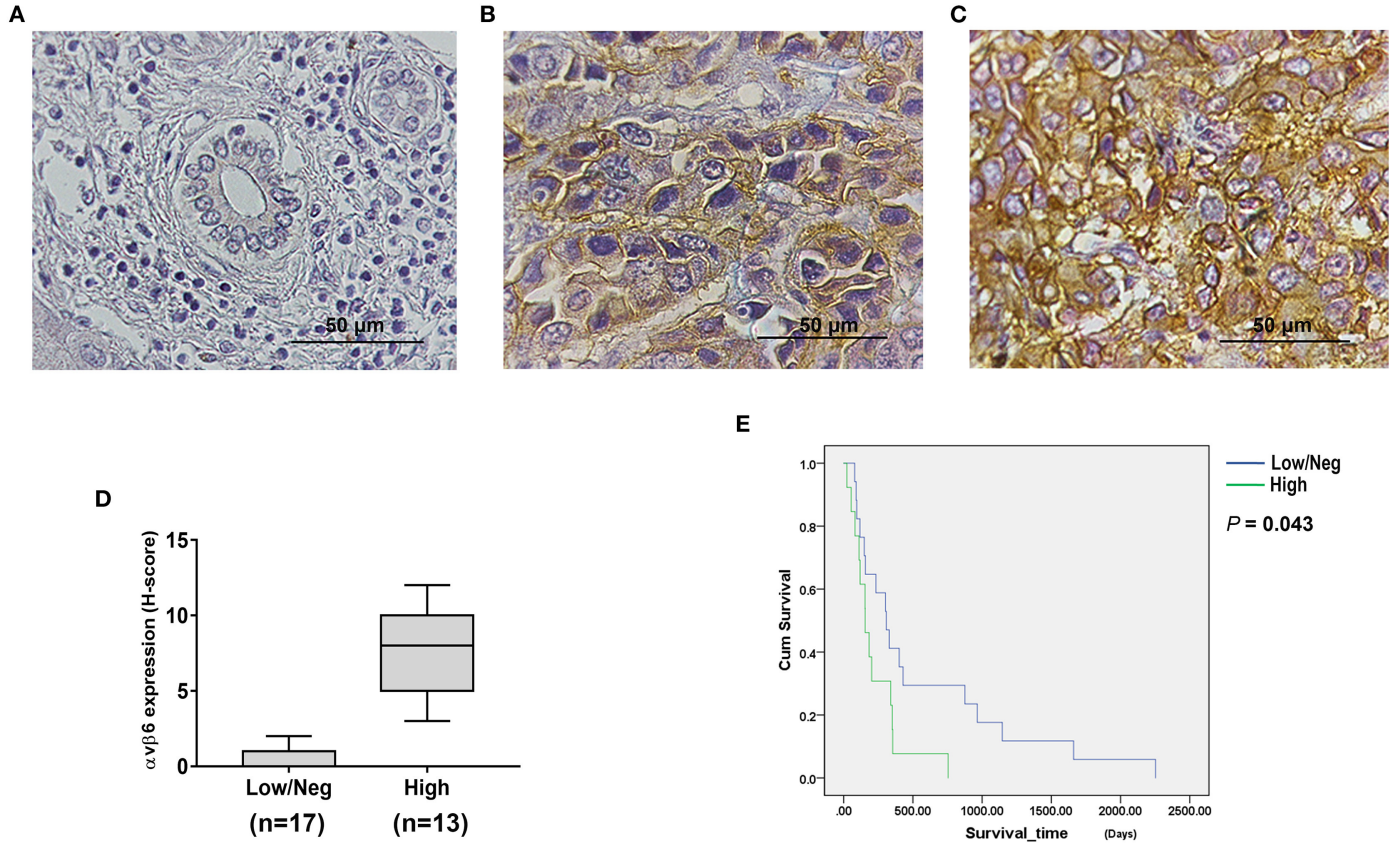
Integrin αvβ6 was upregulated in cholangiocarcinoma (CCA) tissues. Immunohistochemical (IHC) staining was performed to examine integrin αvβ6 expression in CCA tissues obtained from liver fluke-associated CCA patients. **(A)** Negative staining of normal bile duct epithelial cells and hepatocytes surrounding the tumor area was observed. Positive staining of the integrin αvβ6 protein was detected in the cytoplasm and membrane of tumor cells with **(B)** low and **(C)** high expression levels. Scale bars represent 50 μm. **(D)** Expression levels of integrin αvβ6 in CCA tissues were summarized as H-scores. **(E)** Cumulative overall survival curves of CCA patients with different expression levels of integrin αvβ6. The cumulative survival time of patients who had high expression levels of integrin αvβ6 was significantly shorter than that of the patients who had low/negative expression levels of integrin αvβ6 (*p*=0.043).

Immunohistochemistry (IHC) staining was performed to examine the expression of integrin αvβ6 in liver fluke-associated CCA tumors and cell lines, including KKU055, KKU100 and KKU213A. The results revealed the presence of integrin αvβ6 in human CCA tissues, whereas the protein was virtually non-existent all non-tumorous tissues (Figure 1). Low to high intensities of staining signals were observed in CCA tissues (Figures 1B,C). The stained protein was located at both the cell membrane and within the cytoplasm of CCA cells. Twenty-three out of 30 (73.3%) human CCA tissue samples showed positive staining, of which 13 tumors (43.3%) showed high level expression. The expression levels of the protein were calculated as H-scores (Figure 1D). Cumulative survival of patients with low/negative and high expression levels of integrin αvβ6 was compared. All patients with CCA had died by the end of the follow-up period. Analysis of survival times showed a significant difference between the CCA patients who had low/negative and those who had high integrin αvβ6 expression levels. The median survival time was 308.0 days (95% confidence interval [CI]: 178.9–437.1) in the patients with low/negative integrin αvβ6 expression level, and 155.0 days (95% CI: 77.5–232.5) in those with high integrin αvβ6 expression level (log-rank test; *p* = 0.043) (Figure 1E).

**Figure 2:**
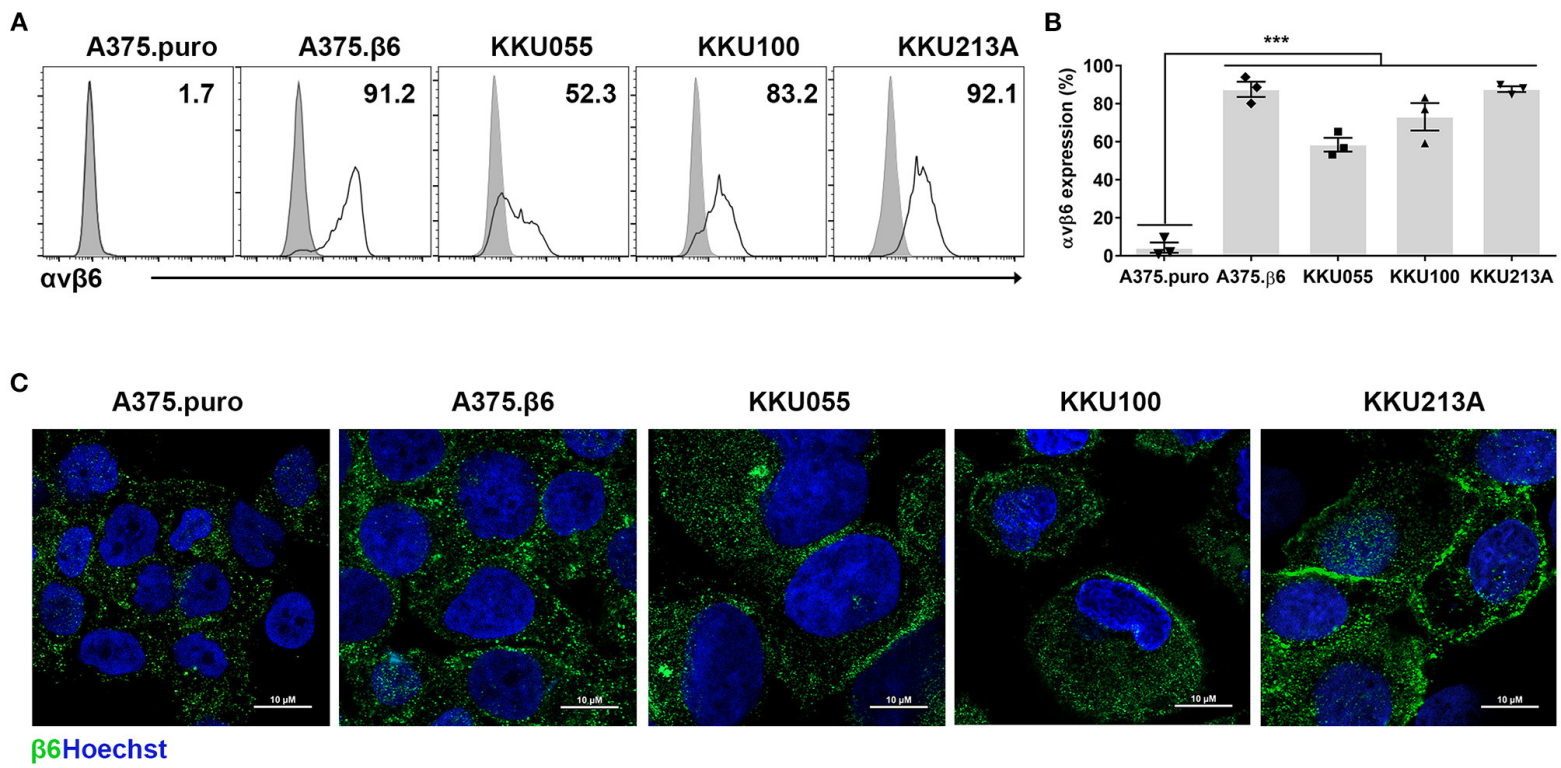
Expression of integrin αvβ6 on cholangiocarcinoma (CCA) cell lines. **(A)** Integrin αvβ6 was stained with anti-integrin αvβ6 mAb in the indicated CCA cell lines and analyzed by flow cytometry. A representative result of flow cytometry analysis demonstrates CCA cell lines stained with isotype-control antibody (gray peaks), and CCA cell lines stained with anti-integrin αvβ6 mAb (white peaks). **(B)** A quantification bar-graph summarizes the data from 3 independent experiments (***p < 0.001). The data with error bar represent mean ± standard error of mean (SEM). Differences between the group of β6-negative A375.puro cells and those of cell lines with positive expression of integrin αvβ6 were analyzed by one-way analysis of variance (ANOVA). **(C)** Immunofluorescence images of CCA cell lines show membranous and granular expression of integrin β6 stained with anti-β6 polyclonal antibody (green) and nuclei staining (Hoechst 33342; blue), and A375.β6 was used as a positive control. Scale bars represent 10 μm.

The expression and localization of integrin αvβ6 was also examined in CCA cell lines. The results showed integrin αvβ6 to be expressed at the cell surface of 58.5 ± 6.2%, 73.2 ± 12.5%, and 87.7 ± 2.6% of KKU055, KKU100, and KKU213A cell lines, respectively (Figures 2A–C). Two known cell lines with negative and positive expression of integrin αvβ6, β6-negative A375.puro cells (4.3±4.7%) and β6-positive A375.β6 cells (87.6±7.0%) (32), were also stained and used as negative and positive controls in further studies.

### Expression of Chimeric Antigen Receptor Targeting Integrin αvβ6 in Lenti-X 293T and Human Primary T Cells

**Figure 3:**
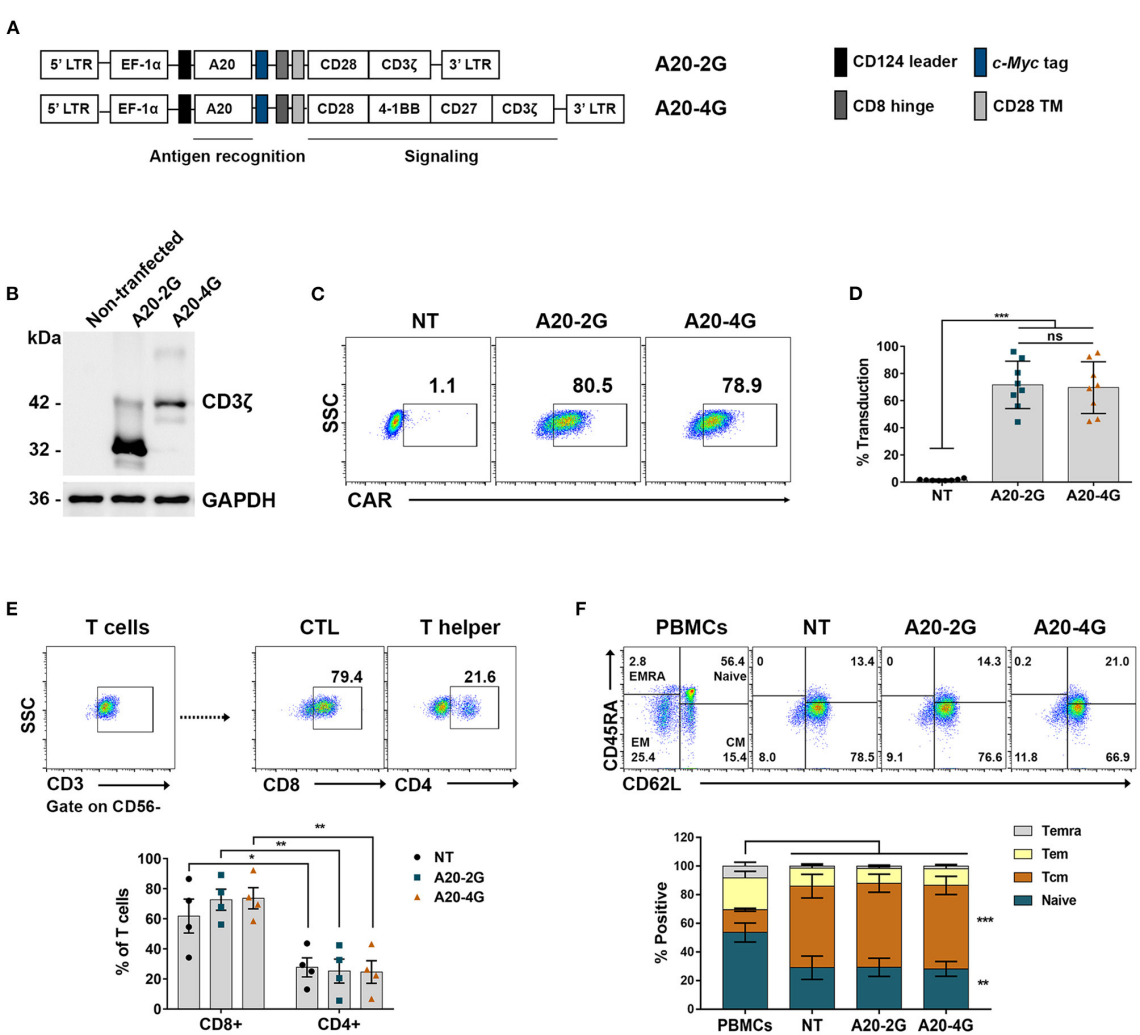
Generation of chimeric antigen receptor (CAR) T cells targeting integrin αvβ6. **(A)** The schematic map demonstrates the A20-2G and A20-4G CAR constructs. The sequence of A20 (integrin αvβ6-targeting ligand) was cloned in-frame into lentiviral vectors linked with the sequences of CD8 hinge, CD28 transmembrane domain (TM), costimulatory domain of CD28/CD3ζ (A20-2G), or CD28/4-1BB/CD27/CD3ζ (A20-4G). **(B)** Expression of A20-2G CAR and A20-4G CAR proteins in Lenti-X 293T cells was detected by immunoblotting analysis using anti-CD3ζ antibody. The data shown represent the mean of three experiments, all of which showed similar results. **(C)** Representative histogram shows expression of A20-2G and A20-4G CAR on the surface of T cells as examined by anti-cMyc FITC-conjugated antibody and flow cytometric analysis. **(D)** Expression of A20-2G or A20-4G CAR on transduced T cells was examined and summarized from eight healthy donors as mean ± standard deviation (SD). Non-transduced (NT) T cells were used as control. **(E)** Representative flow cytometric gating of CD3^+^CD56^−^ T cell population (upper) and summarized data (lower). The phenotypes of CAR T cells were cytotoxic CD8^+^ T cells and helper CD4^+^ T cells. Flow cytometric gating was based on cells stained with isotype-match control antibody. **(F)** The subgroups of A20-2G or A20-4G CAR T cells consisted of CD45RA^−^CD62L^+^ central memory (T_CM_) and CD45RA^+^CD62L^+^ naïve T cells. These data were derived from four healthy donors (ns, non-significant, *p < 0.05, **p < 0.01, ***p < 0.001).

Second- and fourth-generation CAR targeted against integrin αvβ6 (A20-2G CAR and A20-4G CAR) (Figure 3A) were expressed using lentiviral vector. The expression of A20-2G CAR and A20-4G CAR in Lenti-X 293T cells was examined by immunoblotting under reducing conditions using anti- CD3ζ antibody. The results showed that A20-2G CAR and A20-4G CAR were expressed at the predicted sizes of 32 and 43 kDa, respectively (Figure 3B). To generate A20-2G CAR and A20-4G CAR T cells, primary human lymphocytes isolated from a healthy donor were transduced with lentiviruses carrying either A20-2G CAR or A20-4G CAR construct. A representative flow cytometry profile after lentiviral transduction of the lymphocytes is shown in Figure 3C. The median CAR expression of A20-2G CAR was 71.5±17.5%, and A20-4G CAR was 69.6±19.1%, as examined on day five post-transduction (Figure 3D). The phenotypes of CAR T cells were analyzed by flow cytometry, which showed that within the CD3^+^ population, there were significantly more cytotoxic CD8^+^ T cells than helper CD4^+^ T cells in the groups of NT T cells (61.8±11.28% and 27.7±6.3%, *p*=0.038), A20-2G CAR T cells (72.7±7.0% and 25.3±8.0%, *p*=0.004), and A20-4G CAR T cells (73.7±7.0% and 24.6±7.5%, *p*=0.003) (Figure 3E). Furthermore, the generated CAR T cells were enriched in CD45RA^−^CD62L^+^ central memory (T_CM_) and CD45RA^+^CD62L^+^ naïve T cells (Figure 3F). The expression of these markers did not significantly differ between the NT T cells and the A20-2G or A20-4G CAR T cells.

### Anti-tumor Activities of A20-2G and A20-4G CAR T Cells Against Integrin αvβ6-Expressing Cells

**Figure 4:**
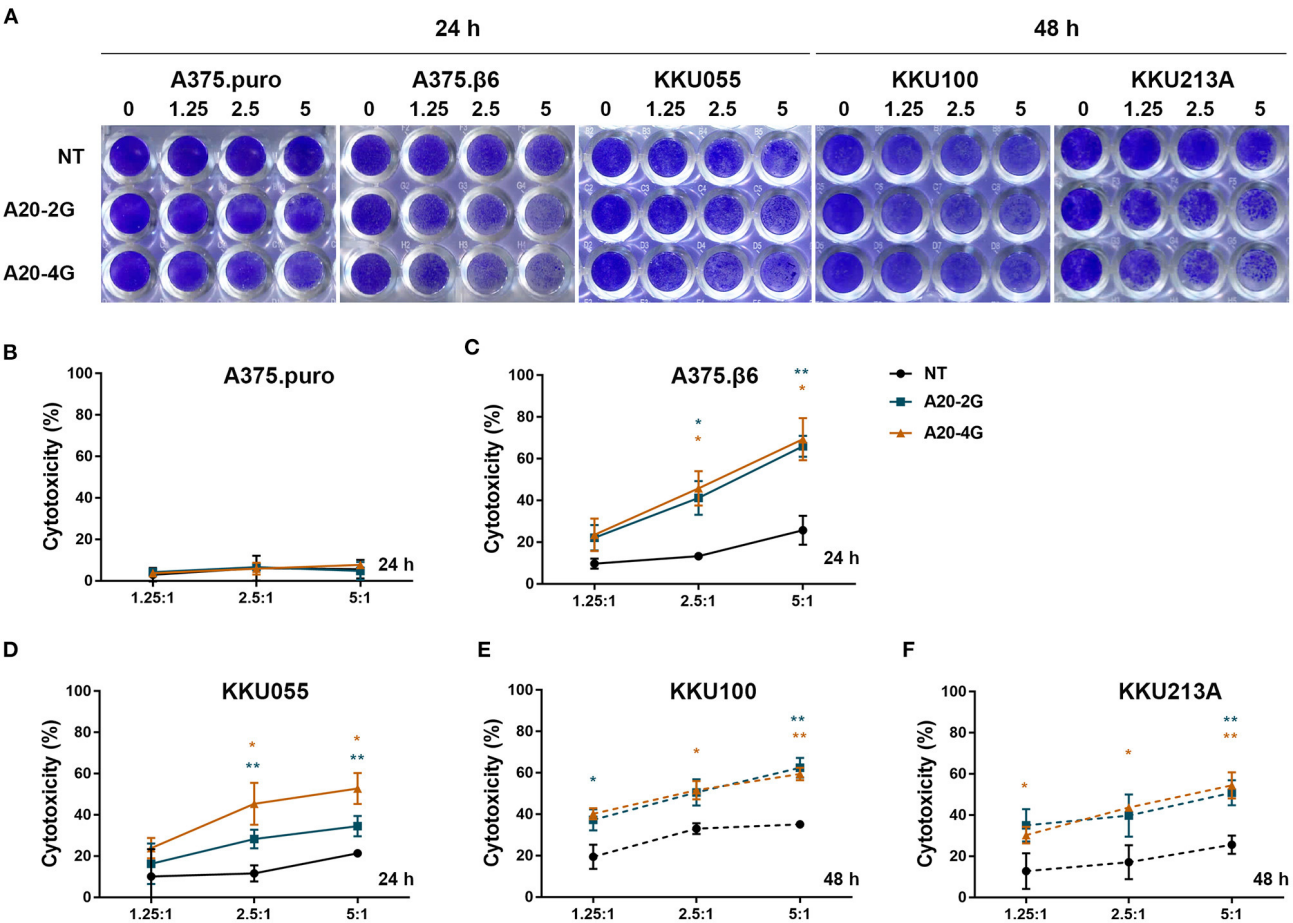
Cytotoxic effects of A20-2G and A20-4G chimeric antigen receptor (CAR)-T cells on integrin αvβ6-negative A375.puro cells, integrin αvβ6-positive A375.β6 cells, and three integrin αvβ6-positive cholangiocarcinoma (CCA) cell lines, including KKU055, KKU100, and KKU213A. The effector CAR T cells were co-cultured with target cells at the indicated effector to target (E:T) ratios (1.25:1, 2.5:1, and 5:1) for 24 h (*solid line*) or 48 h (*dashed line*). **(A)** Cytotoxicity of target cells after co-culturing with effector cells quantified by crystal violet staining assay. Integrin αvβ6-negative A375.puro cell line served as negative control while integrin αvβ6-positive A375.β6 cells was served as positive control. **(B)** Cytotoxic effects of A20-2G and A20-4G CAR T cells on integrin αvβ6-negative A375.puro cells with non-transduced (NT) T cells used as a control for comparison. **(C)** Cytotoxic effects of A20-2G and A20-4G CAR T cells on integrin αvβ6-positive A375.β6 cells with NT T cells used as a control for comparison. Cytotoxic effects of A20-2G and A20-4G CAR T cells on integrin αvβ6-positive KKU055 **(D)**, KKU100 **(E)**, and KKU213A **(F)** cells as compared to the killing activity of NT T cells on the same target cells. Percentage of cytotoxicity relative to the total number of tumor cells alone (set at 0% cytotoxicity) from three independent experiments presented as mean ± standard error of the mean (SEM) (*p < 0.05, **p < 0.01).

The anti-tumor activity of A20-2G and A20-4G CAR T cells against integrin αvβ6-expressing cells was tested by co-culturing these CAR T cells with target cell lines expressing different levels of integrin αvβ6 at the indicated E:T ratio (Figure 4), and using NT cells as control T cells. After co-culture and removal of effector cells, crystal violet solution was added to stain the remaining target cells (Figure 4A). The results revealed that both A20-2G and A20-4G CAR T cells exhibited strong cytotoxic effects in a dose-dependent manner, and both had higher cytotoxic effects than NT T cells. At an E:T ratio of 5:1 and an incubation time of 24 h, the killing activity of A20-2G and A20-4G CAR T cells on integrin αvβ6-negative A375.puro cells was very low (Figure 4B), and the killing activity of A20-2G and A20-4G CAR T cells on integrin αvβ6-positive A375.β6 cells was as high as 65.9±5.06% and 69.4±10.1%, respectively, compared to the killing activity of NT T cells on A375.β6 cells, which was 25.7±6.9% (*p* < 0.05) (Figure 4C). Killing effects were also observed when A20-2G and A20-4G CAR T cells were co-cultured with KKU055 cells (34.6±2.9% and 52.8±7.5%, respectively) compared to the killing activity of NT T cells on KKU055 cells, which was 21.3±0.8% (*p* < 0.05) (Figure 4D). At an E:T ratio of 5:1 and an incubation time of 24 h, no significant cytotoxicity was observed in assays with the KKU100 and KKU213A cells (data not shown). However, after co-culturing for 48 h, the killing activity of A20-2G and A20-4G CAR T cells on KKU100 cells was 62.4±4.7% and 59.4±3.0%, respectively, compared to the killing activity of NT T cells on KKU100 cells, which was 35.1±0.4% (*p* < 0.05) (Figure 4E). Lastly, the killing activity of A20-2G and A20-4G CAR T cells on KKU213A cells was 50.9±6.1% and 54.5±6.3%, respectively, compared to the killing activity of NT T cells on KKU213 cells, which was 25.6±4.5% (*p* < 0.05) (Figure 4F).

### Anti-tumor Activities of A20-2G and A20-4G CAR T Cells Against Three Dimensional CCA Spheroids Expressing Integrin αvβ6

**Figure 5:**
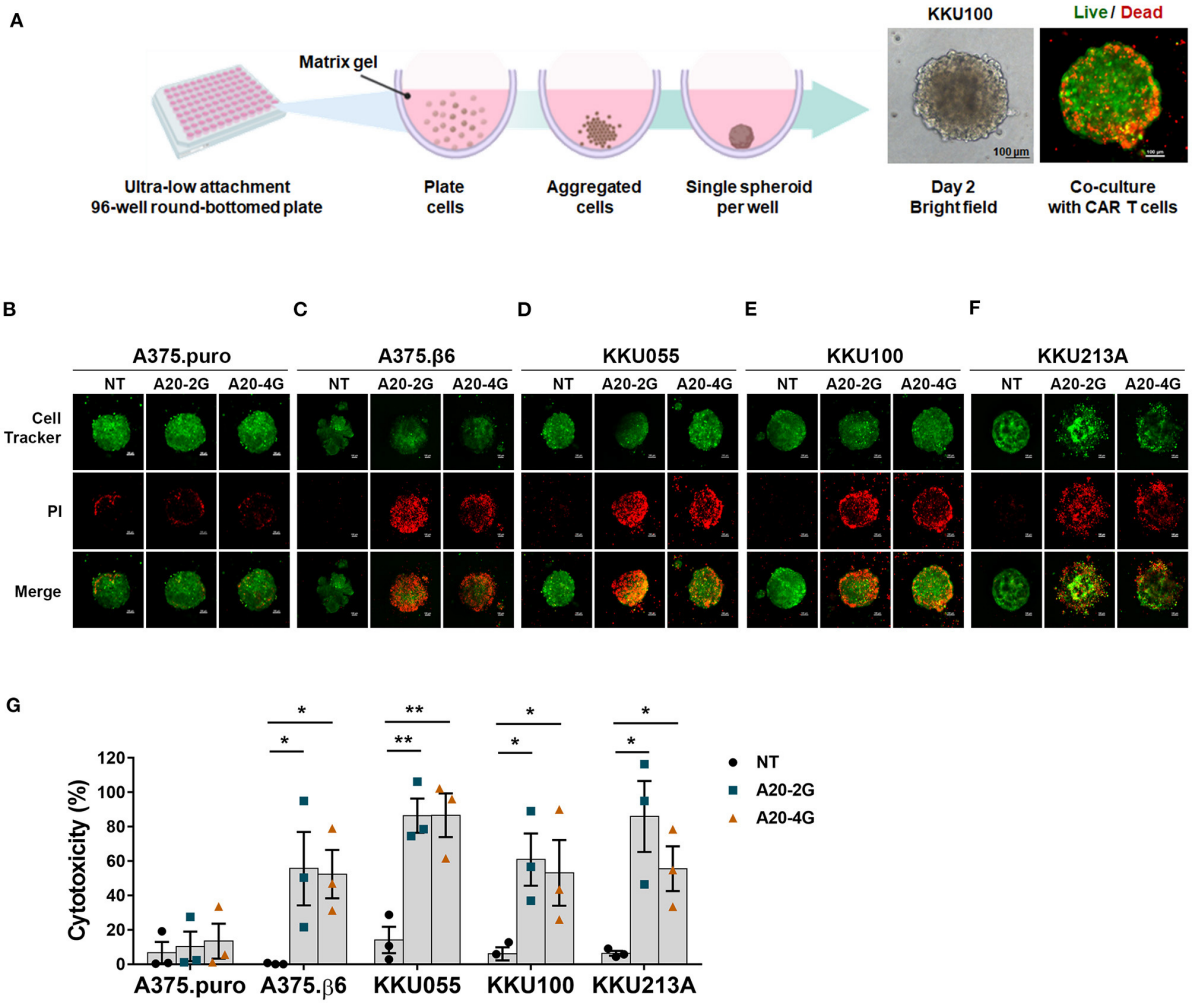
A20-2G and A20-4G CAR T cells mediated killing of CCA tumor spheroids. **(A)** Schematic diagram of spheroid forming in a round-bottomed plate. Representative images on the right show bright filed of a spheroid formed by 2 × 10^3^ cells for 48 h and a fluorescence image after co-culturing CAR T cells with the spheroid. **(B–F)** Representative images showing cytolytic activities of A20-2G or A20-4G CAR T cells compared to NT cells at an effector to target (E:T) ratio of 5:1 on the spheroids of A375.puro, A375.β6, and CCA (KKU055, KKU100, KKU213A) at 72 h of co-culturing. Dead of green fluorescence-labeled cells were visualized by PI uptake (Red). **(G)** Histogram shows cytotoxic activities of NT cells, A20-2G CAR T cells, and A20-4G CAR T cells on tumor cells determined by PI incorporation of dead cells within spheroids. Note that lower level of the percentage of cytotoxicity of A20-4G CAR T cells for KKU213A resulted from more CCA cell lysis. The bar graphs represent mean ± standard error of the mean (SEM) (n = 3; *p < 0.05, **p < 0.01).

Anti-tumor activities of A20-2G and A20-4G CAR T cells against three-dimensional CCA spheroids expressing integrin αvβ6, which appeared like solid tumor in the human body, were also examined. The A20-2G or A20-4G CAR T cells were co-cultured with tumor spheroids for 3 days, and then dead tumor cells were stained by propidium iodide (PI). Confocal microscopy and computerized image processing were employed to locate area of tumor spheroid, and cancer cell death was quantified by integrating PI fluorescence intensity in the spheroid area (Figure 5A). The results revealed that both A20-2G and A20-4G CAR T cells specifically killed the αvβ6-positive A375.β6 and CCA spheroids (KKU055, KKU100, and KKU213A) (Figures 5B–G). While A20-2G and A20-4G CAR T cells showed very weak cytotoxicity on αvβ6-negative A375.puro spheroids, they exhibited more potent cytotoxicity against A375.β6 spheroids, compared to NT T cells (55.6±21.4% or 52.4±14.1% vs. 0.3±0.2%; *p* < 0.05). Killing effects were observed when A20-2G and A20-4G CAR T cells were co-cultured with KKU055 spheroids (86.4±1.0% and 86.64±12.7%, respectively), compared to that of NT T cells on KKU055 spheroids, which was 14.0±7.7% (*p* < 0.01). The killing activity of A20-2G and A20-4G CAR T cells on KKU100 spheroids was 60.9±15.2% and 53.1±19.1%, respectively, compared to that of NT T cells on KKU100 spheroids, which was 6.0±3.8% (*p* < 0.05). Killing effects were also observed when A20-2G and A20-4G CAR T cells were co-cultured with KKU213A spheroids (85.9±20.7% and 55.6±13.0%, respectively), compared to that of NT T cells on KKU213A spheroids, which was 6.4±1.4% (*p* < 0.05). These data indicated that CAR T cells targeting integrin αvβ6 could infiltrate into three-dimensional spheroids and kill cancer cells expressing integrin αvβ6.

### Production of Interferon-γ in and Proliferation of A20-2G and A20-4G CAR T Cells After Co-culturing With Integrin αvβ6-Expressing Target Cells

**Figure 6:**
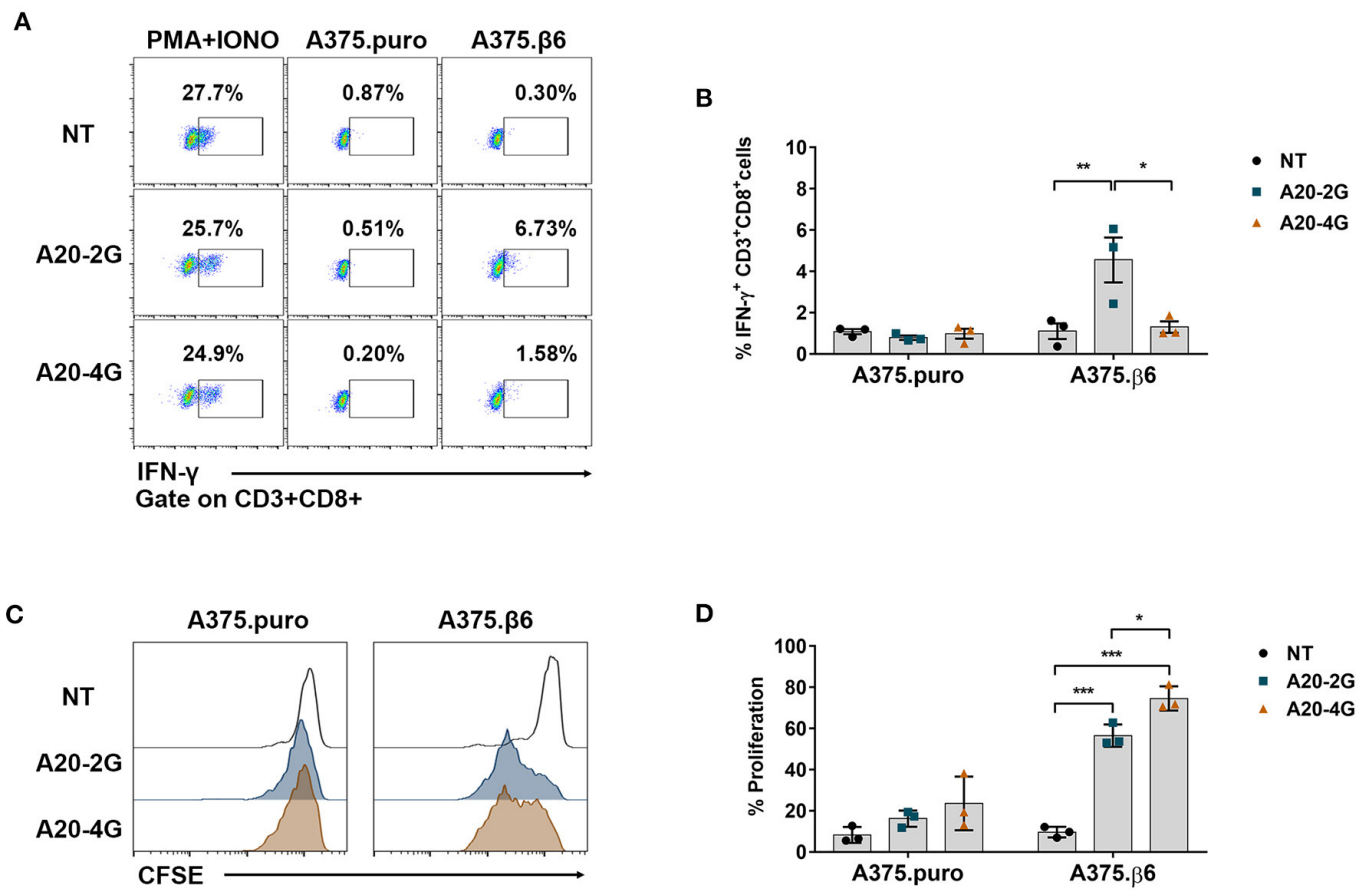
Production of interferon-γ (IFN-γ) and cell proliferation non-transduced (NT) T cells, A20-2G CAR T cells, and A20-4G CAR T cells after co-culture with integrin αvβ6-expressing target cells. **(A)** IFN-γ production was examined by intracellular cytokine staining and flow cytometry analysis in NT T cells, A20-2G CAR T cells, and A20-4G CAR T cells after co-culturing with integrin αvβ6-negative A375.puro or integrin αvβ6-positive A375.β6 cells. **(B)** IFN-γ expression from three individual healthy donors. Data are presented as mean ± standard error of the mean (SEM). **(C)** Proliferation of NT cells, A20-2G CAR T cells, and A20-4G CAR T cells was examined after co-culturing for 3 days with integrin αvβ6-negative A375.puro or integrin αvβ6-positive A375.β6 cells at an effector to target (E:T) ratio of 5:1 without addition of exogenous cytokine. **(D)** Summary data of T cell proliferation derived from 3 individual healthy donors. (*p < 0.05, **p < 0.01, ***p < 0.001).

Production of interferon-γ (IFN-γ) in A20-2G and A20-4G CAR T cells was examined after these CAR T cells were co-cultured with integrin αvβ6-negative A375.puro or integrin αvβ6-positive A375.β6 cells at an E:T ratio of 5:1 and an incubation time of 6 h. As positive control, the CAR T cells were activated using phorbol-12-myristate-13-acetate (PMA) and ionomycin (IONO) in the presence of brefeldin A. In all cases, production of IFN-γ in CD8^+^ T cell population was examined by intracellular cytokine staining and analyzed by flow cytometry (Figure 6A). When NT T cells, A20-2G CAR T cells, and A20-4G CAR T cells were co-cultured with integrin αvβ6-negative A375.puro cells, the production of IFN-γ was minimal (Figure 6B). When these cells were co-cultured with integrin αvβ6-positive A375.β6 cells, the production of IFN-γ in A20-2G CAR T cells was clearly increased compared to those co-cultured with NT T cells (4.5±1.9 % vs. 1.1±0.7%; *p*=0.0156). However, when A20-4G CAR T cells were co-cultured with integrin αvβ6-positive A375.β6 cells, the production of IFN-γ was not significantly increased (1.3±9.5% vs. 1.1±0.7%; *p* > 0.99), (Figure 6B).

The proliferation of NT cells, A20-2G CAR T cells, and A20-4G CAR T cells was examined after co-culturing for 3 days with integrin αvβ6-negative A375.puro or integrin αvβ6-positive A375.β6 cells at an E:T ratio of 5:1 without addition of exogenous cytokine. Carboxyfluorescein succinimidyl ester (CFSE) dilution of proliferating NT and CAR T cells was analyzed on day 3. The results showed that, after co-culturing with integrin αvβ6-negative A375.puro cells, neither A20-2G CAR T cells nor A20-4G CAR T cells showed significant difference of proliferation, compared with NT T cells (16.2±2.2% or 23.6±7.5% vs. 8.3±2.3%, *p*>0.05). After co-culturing with integrin αvβ6-positive A375.β6 cells, A20-2G CAR T cells proliferated to a significantly greater extent than NT T cells (56.5±5.5% vs. 9.6±2.6%, respectively; *p* < 0.001). Surprisingly, after co-culturing with integrin αvβ6-positive A375.β6 cells, A20-4G CAR T cells proliferated to an even greater extent than NT T cells (74.6±5.9% vs. 9.6±2.6%, respectively; *p* < 0.001) (Figures 6C,D).

## Discussion

A more effective treatment for patients with advanced unresectable/metastatic CCA is urgently needed. Adoptive T cell therapy using CAR T cells has provided promising outcomes against hematological malignancies (33), and its potential for treatment of solid cancers is being extensively investigated. The results of the present study provide evidence that A20-2G and A20-4G CAR T cells targeting integrin αvβ6 protein are efficient in killing CCA cell lines, which indicates their potential for treatment of CCA.

A few candidate target antigens are currently being investigated in clinical trials of CCA (34). The integrin αvβ6 protein is overexpressed in several solid tumors, but it is only minimally expressed in normal tissues (29). Here, we show that integrin αvβ6 represents a novel therapeutic target antigen for CAR T cell immunotherapy in patients with CCA. We initially examined the expression of the integrin αvβ6 protein in liver fluke-associated CCA tissues by IHC staining. We found that 73.3% of tumor samples from the Thai patients with this type of CCA had increased expression of integrin αvβ6 (Figure 1). Expression was highly specific to CCA cells compared to adjacent non-malignant biliary epithelia that had an undetectable level of integrin αvβ6. These results are consistent with those observed in CCA tissues from other ethnic groups, including Swiss (26), Japanese (21), and Chinese populations (22). Notably, the survival time of CCA patients who had low/negative integrin αvβ6 protein expression was significantly longer than the survival time of those who had high integrin αvβ6 expression (Figures 1D,E). Integrin αvβ6 has been reported to promote resistance of CCA cells to cisplatin-induced apoptosis (22), which indicates that it should be targeted using other therapeutic approaches, such as immunotherapy. In this study, we also reported the expression of integrin αvβ6 on the surface of patient-derived CCA cell lines, including KKU055, KKU100, and KKU213A cells (Figure 2). We then generated CAR T cells targeting integrin αvβ6 and tested their anti-tumor function in these cell lines. The expression level of the target antigen was reported to affect CAR T cell functionality (35). Thus, we selected a panel of CCA cell lines with different expression levels of integrin αvβ6 to demonstrate the effectiveness of CAR T cells specific to this target antigen.

In previous clinical trials involving patients with B cell malignancy, CAR T cells containing either CD28 or 4-1BB presented different properties, but they showed a similar antitumor response. CD28-based CAR T cells were rapidly activated and their cytolytic activities were enhanced; however, their persistence was short (<3 months) (36). In contrast, 4-1BB-based CAR T cells demonstrated slow response and exhaustion, but their survival was longer (>1 year) (37). The findings of previous studies suggested that complete treatment response required persistence of CAR T cells (37, 38). Accordingly, a CD27 signaling domain was combined in our 4G CAR design to further support the activation, proliferation, and survival of CAR T cells *in vitro* and *in vivo* (39). In our study, we generated the A20-2G CAR construct containing A20/CD28/CD3ζ to be a control, while the A20-4G CAR construct comprising A20/CD28/4-1BB/CD27/CD3ζ (Figure 3A) was designed to combine the favorable properties of CD28, 4-1BB, and CD27. These two constructs were expressed using a self-inactivating lentiviral vector system. The A20-2G and A20-4G CAR proteins of the predicted size could be detected in Lenti-X 293T cells (Figure 2B). Moreover, A20-2G and A20-4G CAR T cells were successfully generated using T cells isolated from eight healthy donors. The expressions of the A20-2G and A20-4G CARs were 71.5±17.5% and 69.6±19.1%, respectively (Figures 3C,D). The final products contained cytotoxic T cells (CD3^+^CD56^−^CD8^+^) with a CD45RA^−^CD62L^+^ phenotype as major populations (Figures 3E,F). This phenotype was reported to support cancer immune surveillance, long-term expansion, and persistence *in vivo* (40). However, the cell phenotypes did not differ between A20-2G and A20-4G CAR T cells compared to NT T cell control, which suggests that their phenotypes may depend on the manufacturing process that we undertook using PHA-L activation and T cell culture in media containing IL-2, IL-7, and IL-15, which were reported to promote cytotoxic T cell memory phenotype (40, 41).

Our data demonstrates that while the A20-2G and A20-4G CAR T cells had minimal cytolytic activity against αvβ6-negative A375.puro cells, the two CAR T cell populations were clearly able to kill αvβ6-positive A375.β6 cells and CCA cells in an E:T ratio-dependent manner (Figure 4), which indicates their specific killing ability. Additionally, both A20-2G and A20-4G CAR T cells killed KKU055 cells within 24 h, which was faster than the time it took for them to kill KKU100 and KKU213A cells within 48 h. It should be noted that high levels of integrin αvβ6 expression on CCA cells did not correlate with high levels of effector activities of the two CAR T cells. The most likely explanation is that CCA cells expressing the integrin αvβ6 protein may also express immune checkpoint molecules to suppress CAR T cell function. The upregulation of PD-L1 in CCA cells has been reported (42), and it can induce T cell exhaustion via the engagement of PD-1 on CD8^+^ T cells (43, 44). A combination of CAR T cells and immune checkpoint inhibitor may improve effector functions of CAR T cells for treatment of CCA (45).

The traditional two-dimensional (2D) culture model based on the growth and proliferation of monolayer cells might not represent the condition with the presence of cell-cell and cell-extracellular matrix interactions. Thus, three-dimensional (3D) CCA spheroids that appeared like solid tumor were generated to evaluate anti-tumor activities of A20-2G and A20-4G CAR T cells. The results showed that the two CAR T cells could infiltrate into the spheroid and displayed potent anti-tumor activities, as demonstrated by dead cancer cells in the spheroids stained by propidium iodide (PI) (Figure 5). In the CCA patients, T cell infiltration in the CCA tissue is a positive outcome predictor (46). A study using 3D culture system revealed that gene expression in this culture system was much closer to clinical expression profiles than those observed in the 2D culture system (47), indicating the suitability of the 3D culture system for preclinical studies.

After co-culturing with αvβ6-positive cells, A20-2G CAR T cells produced greater levels of IFN-γ than A20-4G CAR T cells (4.5±1.9% vs. 1.1±0.7%; *p*=0.0156) (Figures 6A,B). This may be an advantage of A20-4G CAR T cells since clinical studies of the 4G CAR T cells targeting CD19 found that low levels of IFN-γ might be beneficial to limit CAR T cell-mediated cytokine release syndrome (CRS) (10, 15), which was often observed in patients who received CAR T cell therapy (48, 49). Furthermore, A20-4G CAR T cells showed a higher proliferation rate than A20-2G CAR T cells (74.6±5.9% vs. 56.5±5.5%, *p*=0.0175) (Figures 6C,D). This higher proliferation rate may result from the incorporation of CD27 into the A20-4G CAR construct because its signaling is known to typically promoting T cell proliferation (38). Our data may also suggest the addition of 4-1BB/CD27 into the A20-4G CAR construct to be more effective than the addition of only CD28 into the A20-2G CAR construct. Thus, A20-4G CAR T cells possibly offer better benefits than A20-2G CAR T cells for CCA treatment because they may cause less severe CRS response and they have a higher proliferation rate.

A previous study reported the use of CAR T cell immunotherapy to treat a patient with advanced unresectable/metastatic CCA that proved resistant to chemotherapy and radiotherapy (34). Sequential infusions of CAR T cell therapies targeted against EGFR and CD133 induced partial response (PR) for 8.5 months and 4.5 months, respectively. That study showed that CAR T cell therapy targeting two or more antigens is feasible for resolving the problem of tumor heterogeneity in CCA (34). Thus, the generation of CAR T cells specific to integrin αvβ6 and other tumor-associated antigens for treatment of CCA warrants further study. Moreover, since CCA is characterized as having desmoplastic stroma and an immune suppressive tumor microenvironment (TME), the combination of CAR T cell therapy with other treatment modalities, such as chemotherapy using gemcitabine/cisplatin drugs (5), immune checkpoint blockade (45), and/or FGFR inhibitor (6), may overcome immune escape mechanisms of CCA.

Here, we report integrin αvβ6, which is upregulated in CCA tissues, to be a promising target antigen for adoptive T cell therapy of CCA. A20-2G and A20-4G CAR T cells targeting integrin αvβ6 were successfully generated, and both were found to effectively kill αvβ6-positive CCA cells in both monolayer cell and spheroid culture systems. The A20-4G CAR T cells were found to be superior to the A20-2G CAR T cells concerning their higher proliferative potential and lower cytokine production. Thus, the A20-4G CAR T cells warrant further study for their therapeutic potential against CCA.

## Data Availability Statement

The original contributions presented in the study are included in the article/supplementary material, further inquiries can be directed to the corresponding author/s.

## Ethics Statement

The studies involving human participants were reviewed and approved by Siriraj Institutional Review Board of the Faculty of Medicine Siriraj Hospital, Mahidol University, Bangkok, Thailand. The patients/participants provided their written informed consent to participate in this study.

## Author Contributions

NP designed and performed experiments, analyzed data, interpreted results, and prepared manuscript. CS and KS partly performed experiments and analyzed data. MJ and PY conceptualized, managed, and supervised the study. NP, CS, KS, TC, JS, SW, JM, MJ, and PY provided materials and reagents, designed experiments, interpreted results, and edited the manuscript. All authors read and approved the submitted version.

## Conflict of Interest

The authors declare that the research was conducted in the absence of any commercial or financial relationships that could be construed as a potential conflict of interest.
